# Platelet activation and short-term rifaximin therapy in decompensated cirrhosis: a pilot randomized controlled study

**DOI:** 10.1007/s11739-026-04399-7

**Published:** 2026-05-29

**Authors:** Simone Di Cola, Francesca Maiorca, Lucia Stefanini, Annamaria Sabetta, Ludovica Lombardi, Tania D’Amico, Lucia Lapenna, Valentina Castellani, Giulio Francesco Romiti, Roberto Cangemi, Oliviero Riggio, Valeria Raparelli, Manuela Merli, Stefania Basili

**Affiliations:** 1 Department of Translational and Precision Medicine, Sapienza University of Rome, Rome, Italy; 2 Department of Medical and Surgical Sciences and Biotechnologies, Sapienza University of Rome, Latina, Italy; 3 Department of Wellbeing, Health and Environmental Sustainability, Sapienza University of Rome, Rieti, Italy

**Keywords:** Neutrophil extracellular traps, Endotoxin, Gut dysbiosis, Hemostasis, Inflammation

## Abstract

The concept of "rebalanced hemostasis" has improved our understanding of the coexistence of bleeding and thrombosis in cirrhosis. This balance, including changes of platelet function, is easily disrupted by inflammation, through bacterial gut translocation. The modulation of platelet activation through non-absorbable antibiotics remains unexplored. A cross-sectional analysis between patients with decompensated cirrhosis (Child–Pugh, CP, classes B and C) versus age-matched controls without cirrhosis and cardiometabolic diseases was performed to characterize the platelet phenotype and assessed short-term rifaximin effects. Platelet phenotyping, soluble myeloid cells TREM-like transcript-1 (sTLT-1), serum lipopolysaccharides (LPS), inflammatory cytokines, and neutrophil extracellular traps (NETs) markers were quantified. Decompensated cirrhosis patients were randomized to either rifaximin 550 mg BID or placebo for 14 days. Primary outcome was the post-treatment LPS change. Twenty-one patients with decompensated cirrhosis (mean age 59 ± 9 years; 65% male sex, 70% alcohol etiology; mean CP, 7.7 ± 2.2) were matched with sixteen controls (mean age 60 ± 13 years; 37,5% male sex). Patients displayed higher levels of LPS, inflammatory cytokines (IL-1β, IL-6, IL-18, IL-23), and NETs than controls. Platelets from patients with cirrhosis showed significantly lower surface expression of TLT-1 and higher levels of cleaved sTLT-1 (i.e., platelet activation in vivo). Rifaximin did not significantly reduce LPS, inflammatory cytokines, NETosis, and platelet activation. Patients with decompensated cirrhosis exhibit a distinct activation state of platelets with low-grade systemic inflammation and NETosis. Short-term rifaximin failed to reverse these changes, suggesting that longer or combined interventions targeting LPS may be required to rescue platelet dysfunction.

**Graphical abstract:**

**Figure:**
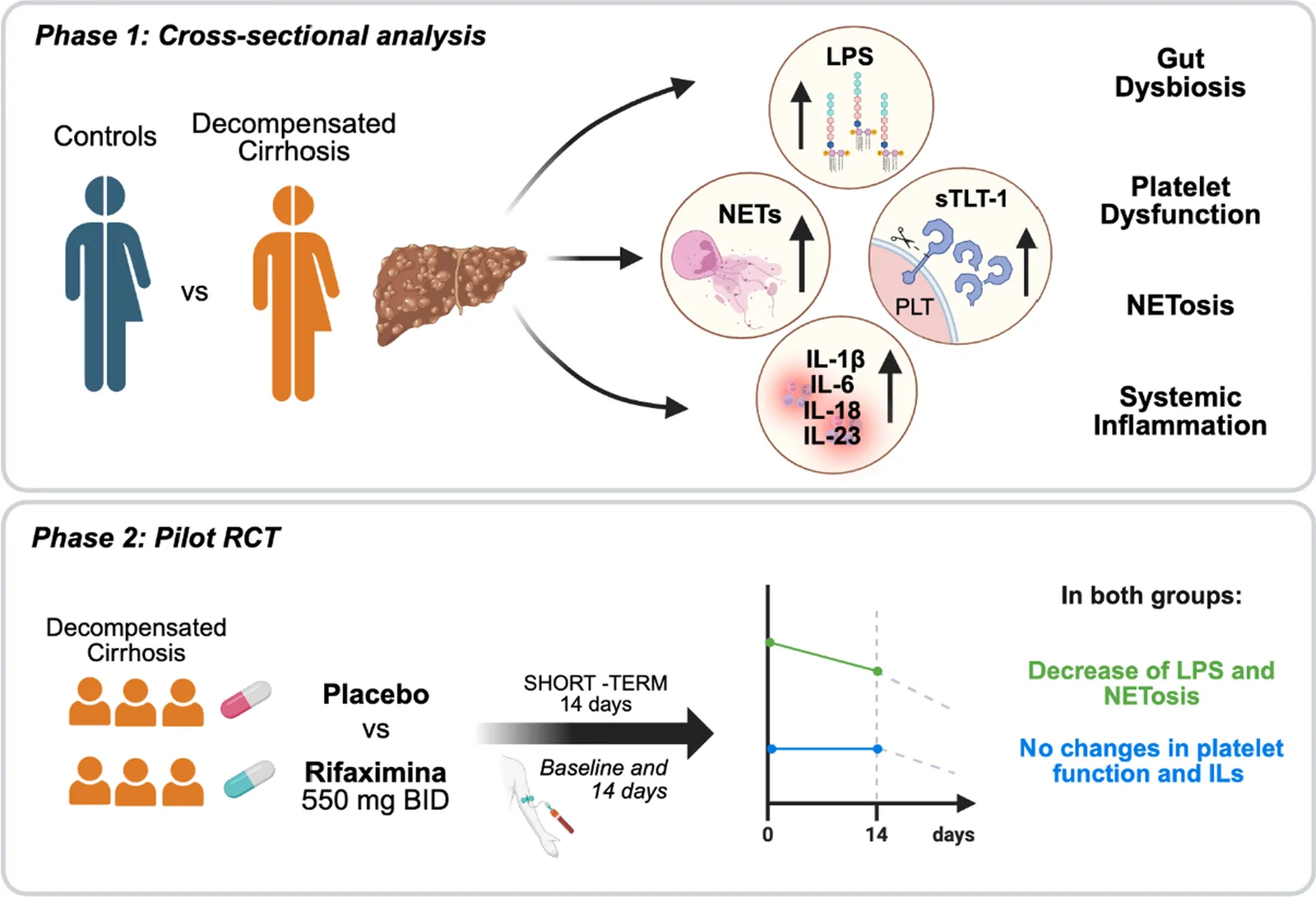
Platelet Activation and Short-term Rifaximin Therapy in Decompensated Cirrhosis. Phase 1: Cross-sectional analysis between decompensated cirrhosis patients and sex- and age-matched controls. Patients displayed higher levels of circulating LPS, inflammatory cytokines (specifically, IL-1β, IL-6, IL-18, IL-23), and NETs than controls. Platelets from patients with cirrhosis showed significantly lower surface expression of the platelet-specific receptor TLT-1 and higher levels of cleaved sTLT-1. Phase 2: Pilot randomized controlled trial (RCT) in patients with decompensated cirrhosis were randomized to either rifaximin 550 mg BID or placebo for 14 days. The short term treatment with rifaximin did not significantly reduce LPS, inflammatory cytokines, NETsosis and platelet activation.

**Supplementary Information:**

The online version contains supplementary material available at https://doi.org/10.1007/s11739-026-04399-7.

## Introduction

In recent years there has been a paradigm shift in the management of cirrhosis. Several lines of evidence have suggested that the clinical coexistence of bleeding and thrombotic events in patients with cirrhosis is due to a state of “rebalanced hemostasis.” This alternative state is more susceptible to disturbance by a number of trigger events, commonly experienced by patients, such as acute kidney injury, bacterial infection, and acute on chronic liver failure [1–3].

Previous work showed that patients with cirrhosis experience a state of low-grade inflammation, mainly sustained by increased levels of bacterial lipopolysaccharides (LPS) [4–6]. Endotoxemia results from impaired intestinal permeability with increased translocation of gut-derived metabolites and bacteria, which is more pronounced in the advanced stages of disease [7, 8]. Notably, LPS has also been implicated as a major driver of both platelet hyperactivation and procoagulant state in advanced and decompensated cirrhosis [9, 10], leading to the increased incidence of thrombotic events [11, 12]. Indeed, interventions targeting LPS and bacterial translocation have led to a modulation of hemostatic parameters [13] and even to the prevention of portal vein thrombosis (PVT) [14].

Platelets are the first actors in the hemostatic cascade, and thrombocytopenia is a well-known hallmark of cirrhosis. The contribution of dysregulated platelet function to the thrombotic risk of patients with cirrhosis has been less explored over time because of the intrinsic limitations of platelet testing in patients with thrombocytopenia [15]. However, techniques such as flow cytometry now provide the opportunity to study the platelet phenotype and function in patients with low platelet count where other assays (LTA, impedance aggregometry, and point-of-care cartridge-based assays) are seriously compromised or flawed [15].

Thus, this study aimed (i) to better characterize the platelet phenotype exploiting flow cytometry in advanced decompensated cirrhosis and (ii) to test whether acute modulation of low-grade inflammation can help restore the hemostatic balance.

## Materials and methods

### Study design

A two phase-study was planned: cross-sectional phase and pilot randomized controlled trial (RCT).

Consecutive patients with cirrhosis of various causes were recruited after giving written informed consent at the Policlinico Umberto I – Sapienza University of Rome, Italy, between November 2021 and March 2024. Inclusion criteria were as follows: patients aged 18–75 years, with decompensated cirrhosis defined by Child–Pugh class B or C at the time of assessment. Exclusion criteria were as follows: presence of overt infection or sepsis, treatment with systemic or non-absorbable antibiotic, aspirin or other non-steroidal anti-inflammatory drugs, diagnosis of hepatocellular carcinoma (HCC), antidepressant drugs in the previous 30 days, need of transfusion of platelets or plasma within 30 days before the screening period, presence of extrahepatic malignancy, active alcohol intake in the last 6 months, pregnancy or breastfeeding, presence of overt hepatic encephalopathy (HE), gastrointestinal hemorrhage, spontaneous bacterial peritonitis or other concurrent infections during the previous month, human immunodeficiency virus (HIV) infection, chronic renal and/or respiratory insufficiency, severe heart disease, allergy to rifaximin, active post-viral hepatitis requiring or on direct-acting antiviral agents, use of cyclosporine and P-glycoprotein inhibitors, and previous or active intestinal obstruction.

Adults without cirrhosis and cardiometabolic diseases matched by age were recruited after giving written informed consent at the Policlinico Umberto I – Sapienza University of Rome, Italy, as a control group.

After the first cross-sectional study to test platelet function, a pilot RCT, double-blind and placebo controlled, has been performed to test whether an intervention targeting bacterial translocation and low-grade inflammation might modulate platelet function. Patients with decompensated cirrhosis were randomly assigned, in a 1:1 ratio, to receive either 550 mg of rifaximin or placebo BID for 2 weeks or until they discontinued the study drug because of adverse events. At the end of the treatment period, patients were asked to return the blister packs to verify treatment adherence and confirm that all tablets had been taken (as part of Investigational medicinal product (IMP) compliance).

Lactulose was allowed to be co-administered during the trial if the patient had already been receiving it prior to enrollment.

All the concomitant therapies remained unchanged during the 14 day treatment period and the following 30 days of observation phase, except when clinically required. Other antibiotics have been prohibited during the 14 day treatment period and the 30 days observation. Best clinical practice was guaranteed for all patients according to the guidelines for the management of clinical decompensation [16]. Ascites decompensation was classified as grades 1, 2 and 3 according to the guidelines [17], while HE was reported according to the West Heaven classification [18].

As a primary outcome of the pilot RCT, changes in serum bacterial LPS and markers of platelet dysfunction were evaluated after the short-term treatment. The occurrence of safety adverse outcomes was monitored during the interventional phase. At each visit, patients were asked to complete a questionnaire on commonly reported major and minor side effects of rifaximin (Patient Prefer Adherence. 2014; 8: 331–338). Patients were counseled on symptoms and signs, and a telephone number was provided for each subject enrolled in the study. At each visit, each subject underwent a safety laboratory analysis and a full physical examination. Suspected unexpected serious adverse events, treatment-emergent adverse events, and serious adverse events (SAEs) were considered for withdrawal from the study and were reported on a special form.

This study has been conducted according to the principles stated in the Declaration of Helsinki and after approval by the local ethical committee (Ethical Committee number Rif. 4438). This study was designed by academic authors. The Alfasigma Pharmaceutics provided the supply of rifaximin and placebo; nevertheless, it played no role in the design or conduct of the trial, the collection or analysis of the data, or the review or editing of the manuscript. The authors vouch for the completeness and accuracy of the data and for the adherence of the trial to the protocol. No one who is not an author contributed to the writing of the manuscript.

The study protocol was registered on ClinicalTrial.gov (NCT06630572) and on the European Union Clinical Trials Register (EudraCT number 2017–000488-34).

Venous blood samples were collected at the entry and at the end of the treatment as well as after 30 days after drug discontinuation. Clinical and laboratory data were collected at baseline, end of treatment, and 30 days after drug withdrawal, including assessment of ascites and HE, recording of gastrointestinal bleeding, hospitalization, MELD and CP scores, and changes in medical treatment.

### Biological sample collection

On the day of enrollment and after 14 day treatment, venous whole blood was collected in fasting conditions. EDTA-anticoagulated blood was used to determine blood cell counts and the platelet morphologic parameters with a Sysmex XP-300 (Sysmex Corporation). Sodium-citrate anticoagulated whole blood was used within 30 min from blood withdrawal for platelet studies. Serum and plasma were isolated by centrifugation at 2000 g for 20 min and stored at − 80 °C until batch analysis. EDTA-plasma and serum were used, respectively, to measure soluble TLT-1 and LPS levels. Details of experimental methods are reported in Supplementary material.

### Statistical analysis

Categorical variables are expressed as counts and percentages. Continuous variables are reported as mean ± SD or median and interquartile range (IQR). Continuous variables were compared using Student t-tests for normally distributed continuous variables and the Mann–Whitney U test for not-normally distributed ones. To adjust for multiple testing multiple Mann–Whitney U tests were corrected using the Holm-Šídák method. A two-sided *P* value < 0.05 was considered statistically significant. The before–after treatment analyses were performed with Multiple Wilcoxon tests. All analyses were performed using computer software packages (GraphPad Prism 10).

## Results

### Study population

**Fig. 1:**
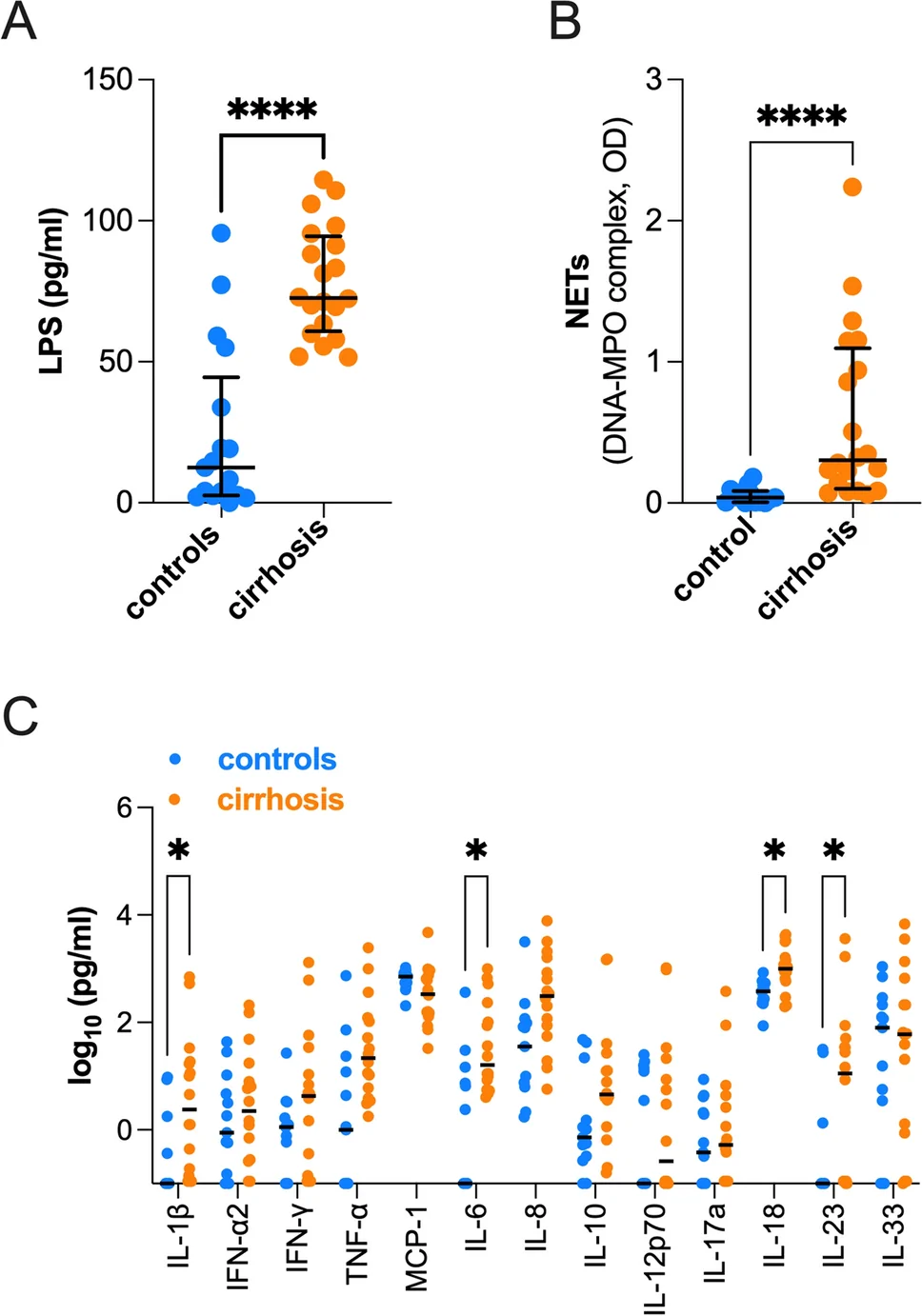
Bacterial translocation and low-grade inflammation in decompensated patients with cirrhotic. Concentration of **A** serum LPS and **B** markers of NETs (DNA-MPO complex) detected by ELISA in decompensated patients with cirrhosis (*n* = 21; cirrhosis, yellow) and age-matched controls without cardiometabolic disease (*n* = 16; control, blue). Statistical significance was determined by Mann–Whitney test; ****p < 0.0001. **C** Inflammatory cytokine profile measured by multiplex bead assay. The scatter dot plot shows the log10 of the cytokine concentration in picograms/milliliter (pg/ml). The line indicates the median value. Statistical significance was determined by multiple Mann–Whitney U test corrected using the Holm-Šídák method; * adjusted *p* < 0.05

During the screening period, 101 patients with Child–Pugh B-C cirrhosis were considered for recruitment, 76 of whom were excluded as they met the following exclusion criteria: 40 patients for concomitant therapy with rifaximin or an active episode of overt HE; 15 patients were excluded for an actual or recent diagnosis of bacterial infection requiring antibiotic therapy; 9 patients were treated with aspirin or NSAIDs; 7 patients had a recent need for transfusion; 4 patients had a diagnosis of HCC; and one patient had concomitant severe heart disease. Four patients were initially screened for enrollment then excluded for consent withdrawal or for unexpected clinical events (one patient withdrew consent after initially agreeing to participate, two developed bacterial infections, and one was diagnosed with HCC during the screening period). We finally enrolled 21 subjects (mean age 59 ± 9 years) with decompensated cirrhosis whose clinical characteristics are shown in Table 1. Patients were compliant with the therapy, as assessed by blister pack inspection at the end of the treatment. Specifically, three patients had five tablets remaining, and four patients had two tablets left. The main etiology of liver disease was alcohol (70%), while hepatitis C and MASLD (Metabolic-Associated Steatotic Liver Disease)-related cirrhosis were both present in 5 (25%) patients. The median MELD and MELD-Na scores were, respectively, 13.0 ± 3.1 and 15.0 ± 3.7 and ascitic decompensation was observed in the majority of the cohort (16 patients, 80%); as a consequence, most patients were on diuretics (90%). A minority of patients received anticoagulants and statins (2 patients each, 5%). Among the entire cohort, 15 patients presented with esophageal varices, including 7 with large varices (F2–F3). Only 4 patients had a history of variceal bleeding. Compared to the control group matched for age (mean age 60 ± 13 years, 37.5% male sex), patients with cirrhosis had elevated serum levels of LPS, increased levels of markers of NETs (*p* < 0.001), and higher levels inflammatory cytokines, most notably IL-1β (adjusted* p* = 0.008), IL-6 (adjusted *p* = 0.017), IL-18 (adjusted *p* = 0.004), and IL-23 (adjusted *p* = 0.01) (Fig. 1).

**Table 1:** Baseline characteristics of patients enrolled in the placebo and treated groups

	Overall cohort (*n* = 21)	Placebo group (*n* = 9)	Rifaximin group (*n* = 12)	*p* value
Age, years, mean (SD)	60 (9)	62 (7)	61 (6)	0.73
Male sex	13 (61)	6 (66.6)	7 (58)	1.00
Alcohol Etiology	14 (66)	7 (77.8)	7 (58)	0.64
BMI	25.1 (6.7)	22.5 (6.3)	26.1 (6.9)	0.22
*Laboratories values*				
Child Pugh score	8 (1.1)	8 (1)	8 (1.5)	1.00
MELD score	13.0 (3.1)	13.0 (3.5)	13.0 (2.8)	1.00
MELD-Na score	15.0 (3.7)	16.0 (3.9)	15.5 (3.8)	1.00
INR	1.48 (0.35)	1.41 (0.25)	1.55 (0.43)	0.36
*Clinical features*				
Ascites decompensation	17 (80)	7 (77.8)	10 (81.8)	1.00
Oesophageal varices	14 (66)	6 (66)	8 (66)	1.00
PVT	2 (9.5)	0	2 (16.6)	0.48
Diabetes	7 (33)	2 (28.6)	5 (41.6)	0.64
Hypertension	3 (14)	1 (14.3)	2 (16)	1.00
*Ongoing therapies*				
Albumin infusion	5 (23)	2 (28.6)	3 (25)	1.00
Diuretics	19 (90)	8 (88.9)	11 (90.1)	1.00
NSBB	12 (57)	6 (66.6)	6 (50)	0.66
Statins	2 (9.5)	1 (14.3)	1 (8.3)	1.00
Anticoagulant	2 (9.5)	1 (14.3)	1 (8.3)	1.00
Lactulose	5 (23.8)	2 (28.6)	3 (25)	1.00
PPI	9 (42.8)	4 (44.4)	5 (41)	1.00

Categorical variables are expressed as number of patients (percentage) and continuous variables are expressed as median (IQR) unless otherwise specified. There were no statistically significant differences between the placebo and treated groups

*BMI* Body Mass Index, *INR* International Normal Ratio, *MELD* Model of End-Stage Liver Disease, *PVT* Portal Vein Thrombosis, *NSBB* Non-selective Beta-blockers, *PPI* Proton Pump Inhibitors

Most of the enrolled patients with cirrhosis (85% vs. 25%; *p* < 0.001) were thrombocytopenic (PLT < 150,000 plt/μl), and had an increased Platelet Distribution Width (PDW) and a significantly reduced immature platelet fraction (IPF) compared to controls (Table 2).

**Table 2:** Hematological profile of enrolled cirrhotic patients and controls

	Variables are expressed as number of patients	Variables are expressed as number of patients	Variables are expressed as number of patients
WBC (× 10^3/ul)	5.3 [4.7–7.3]	5.1 [4.-5.5]	0.08
LYM (× 10^3/ul)	1.95 [1.4–2.2]	0.9 [0.8–1.3]	< 0.01
MXD (× 10^3/ul)	0.5 [0.4–0.6]	0.5 [0.4–0.8]	0.69
NEUT (× 10^3/ul)	3.1 [2.3–4.6]	3.2 [2.6–3.7]	0.63
PLT (× 10^3/ul)	198.5 [142–253]	69.5 [57.2–118]	< 0.01
PDW (fl)	13.4 [12.3–15.8]	15.2 [13.2–17.5]	0.07
MPV (fl)	10.5 [9.8–11.1]	10.8 [9.9–11.2]	0.8
P_LCR (%)	30.1 [25.5–35.5]	33.6 [24.8–36.2]	0.67
IPF (%)	14.1 [4.1–19]	3.3 [0.6–7.1]	< 0.05
RBC (× 10^6/ul)	4.6 [4.3–4.9]	3.7 [3.4–4.4]	< 0.01
HGB (g/dl)	13.5 [12.9–14.5]	11.6 [12.6–10.7]	< 0.01
HCT (%)	41.3 [40.3–44.6]	36.2 [32.8–39.9]	< 0.01
MCV (fl)	89.6 [87.9–92]	93.3 [88.9–98.5]	0.05
MCH ((pg)	29.5 [28.9–30.8]	30.9 [32.1–28.7]	0.11
MCHC (g/dl)	33 [32.3–33.7]	32.7 [33.3–31.4]	0.41
RDW_SD (fl)	45.6 [42.4–48.7]	53.5 [51.4–55.8]	< 0.01
RDW_CV (%)	13.3 [12.1–13.9]	15.9 [14.9–16.5]	< 0.01

Hematological parameters are shown as the median (IQR) unless otherwise stated and statistically differences between the controls and cirrhosis group were indicated in the *p* value column

*HCT* Hematocrit, *HGB* Hemoglobin, *IPF* Immature Platelet Fraction, *LYM* Lymphocyte, *MCH* Mean Corpuscular Hemoglobin, *MCHC* Mean Corpuscular Hemoglobin Concentration, *MCV* Mean Cell Volume, *MPV* Mean Platelet Volume, *MXD* Monocytes, *NEUT* Neutrophils, *PDW* Platelet Distribution Width, *P_LCR* Platelet Large Cell Ratio, *PLT* Platelet, *RBC* Red Blood Cells, *RDW_CV* Red Cell Distribution Width—Coefficient of Variation, *RDW_SD* Red Cell Distribution Width—Standard Deviation, *WBC* White Blood Cell Count

### Characterization of the platelet phenotype in decompensated cirrhosis

**Fig. 2:**
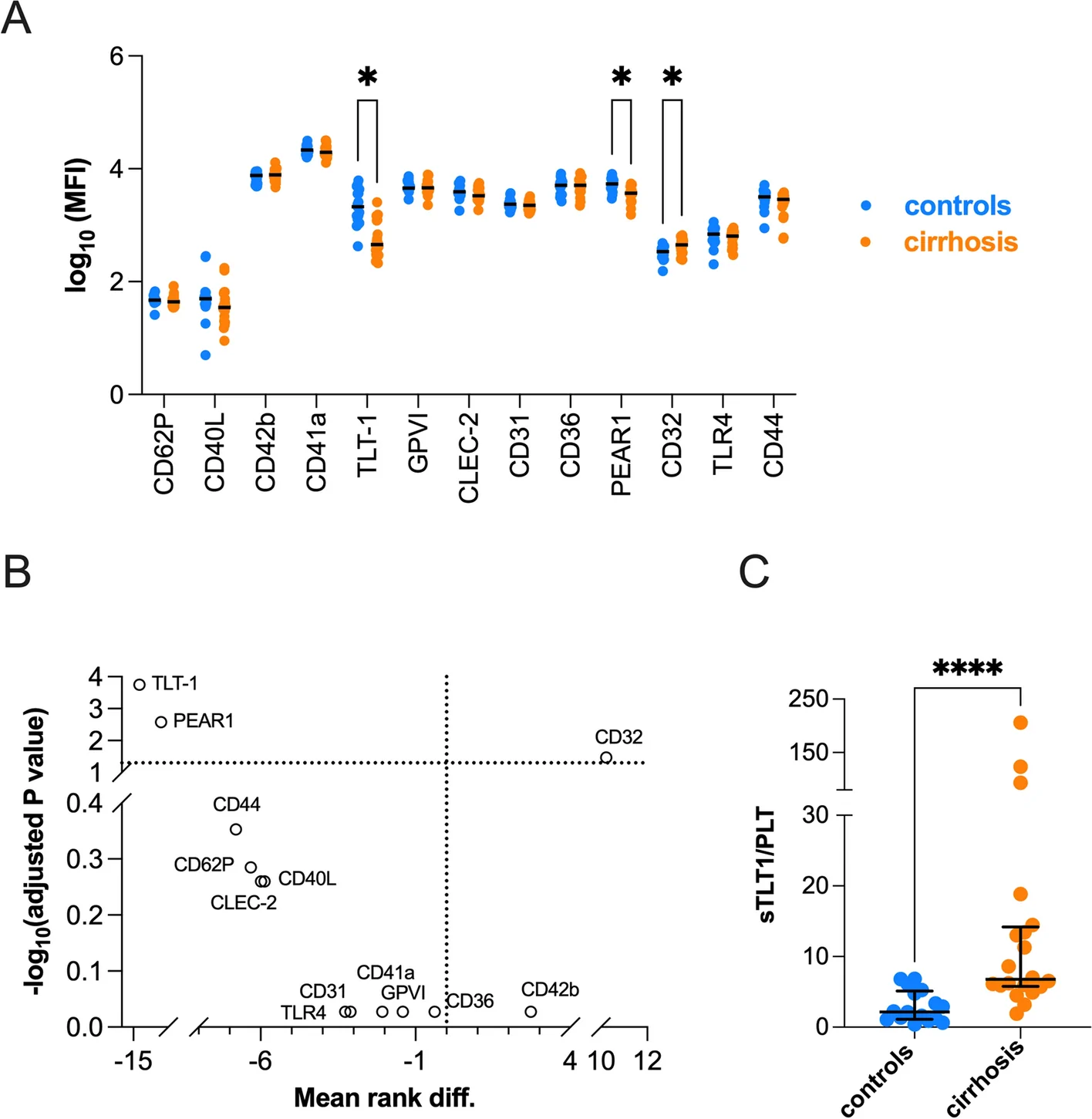
Phenotype of circulating platelets in decompensated patients with cirrhosis. **A** Multiple Mann–Whitney t test of the expression detected by flow cytometry of 13 platelet surface receptor in decompensated cirrhosis patients (*n* = 21; cirrhosis, yellow) compared to age-matched controls without cardiometabolic disease (*n* = 16; CTRL, blue). The graph shows the log10 of the median fluorescence intensity (MFI) of P-selectin (CD62P), CD40 ligand (CD40L), glycoprotein Iba (CD42b), integrin aIIb (CD41a), TREM-like 1 (TLT-1), glycoprotein VI (GPVI), C-Type Lectin Domain Family 1 Member B (CLEC2), PECAM-1 (CD31), CD36, Toll-like receptor 4 (TLR4), Platelet Endothelial Aggregation Receptor 1 (PEAR1), FcγRIIa (CD32), CD44. The line indicates the median value. The Holm-Šídák method was used to adjust for multiple comparisons; * adjusted *p* < 0.05. **B** Volcano plot showing the mean rank difference between patients and controls and the adjusted p value for each platelet feature. Black dots above the horizontal dotted line identify the features that are significantly different; *adjusted *p* < 0.05. (C) Concentration of plasma concentrations of the soluble ectodomain of the TLT1 receptor detected by ELISA, normalized to the platelet count (sTLT1/PLT) of each study participant. Statistical significance was determined by Mann–Whitney test; ****p < 0.0001

Flow cytometric analysis of 13 platelet surface receptors indicated that circulating platelets of patients had a distinct phenotype. Compared to age-matched controls, patients with cirrhosis displayed on the platelet surface lower levels of the Triggering receptor expressed in myeloid cells (TREM)–like transcript 1 (TLT-1) and of the Platelet Endothelial Aggregation Receptor 1 (PEAR1), and higher levels of CD32 (low affinity receptor for IgG) that were statistically significant after adjusting for multiple comparisons (TLT-1: adjusted *p* < 0.001; PEAR1: adjusted p < 0.01; CD32: adjusted *p* < 0.05). Since proteolytic cleavage of TLT-1 is a marker of platelet activation, we also measured the plasma levels of its soluble ectodomain and normalized it to the platelet number to account for the differences between patients and controls. Soluble TLT1 concentration relative to the platelet count (sTLT1/PLT) was significantly elevated in patients with cirrhosis compared to the controls (*p* < 0.0001) (Fig. 2).

### Effect of a short-term therapy with rifaximin in decompensated cirrhosis patients

**Fig. 3:**
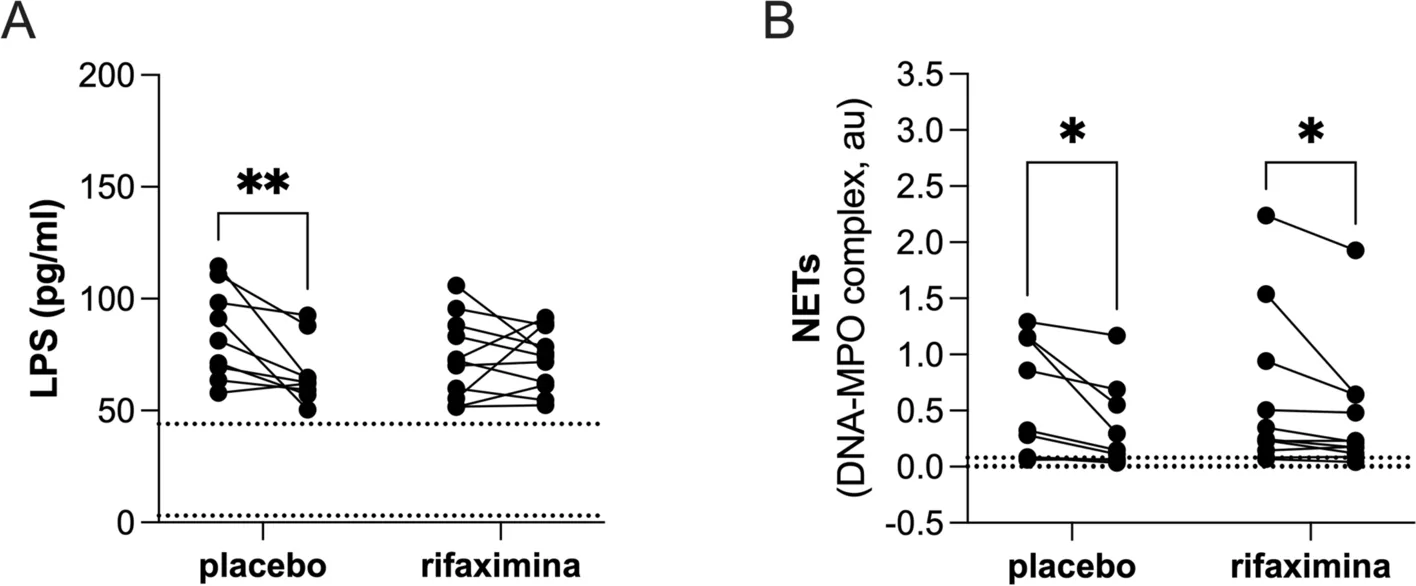
Bacterial translocation and markers of NETs before and after short-term therapy with rifaximin in decompensated cirrhosis patients. Before–after graph of the concentration of **A** serum LPS and **B** markers of NETs (DNA-MPO complex) in individuals decompensated patients with cirrhosis randomized to receive either placebo (n = 9) or 550 mg of rifaximin (*n* = 12) for 14 days. Statistical significance was determined with a Multiple Wilcoxon test; *p < 0.05

To test if the platelet phenotype could be modified by reducing gut dysbiosis, we performed a pilot RCT on the effects of a short-term therapy with rifaximin, a non-absorbable antibiotic. At baseline, no differences in clinical and laboratory values as well as experimental measurements of platelet function were observed between placebo and rifaximin groups (Table 1, Supplementary Figs. 1–2 and Fig. 3). After the treatment period, no differences were observed in the median Child–Pugh, MELD and MELD-Na scores [respectively, 8 (1.1) vs. 7 (1.6) *p* = 0.47; 13.0 (3.1) vs. 13.0 (5.0) *p* = 0.34; 15.0 (3.7) vs. 16.0 (5.8) p 0.39], even when the placebo and rifaximin groups were considered separately. Ascites improved in two patients, one in the rifaximin group and one in the placebo group (from grade 3 to grade 2 and from grade 2 to absence of ascites, respectively), which remained the main clinical complication of the enrolled patients (14 patients, 70% of the entire cohort); only one patient required a large-volume paracentesis during the treatment period, while none of the patients were treated with a Transjugular Porto-Systemic Shunt or any other invasive procedure during the 14-day observation period.

Two episodes of HE were registered, one in each group (grade 2 for the patient in the placebo group and grade 3 and need for hospitalization for the patients in the rifaximin group). There were no episodes of gastrointestinal bleeding or PVT. As previously reported, only one patient (in the rifaximin group) experienced a SAE, specifically a West-Haven grade 3 HE event; this event, which was recorded and reported as per protocol, was not considered to be related to rifaximin therapy but secondary to an ongoing infectious event; however, the drug was discontinued 7 days after the beginning of the treatment.

After 14 days of treatment, LPS levels were marginally reduced compared to baseline, reaching statistical significance only in the placebo group (*p* < 0.05), but not in the treatment group. NETs were reduced in both study groups (*p* > 0.05) (Fig. 3), but the levels of inflammatory cytokines were unchanged (Supplementary Fig. 1).

**Fig. 4:**
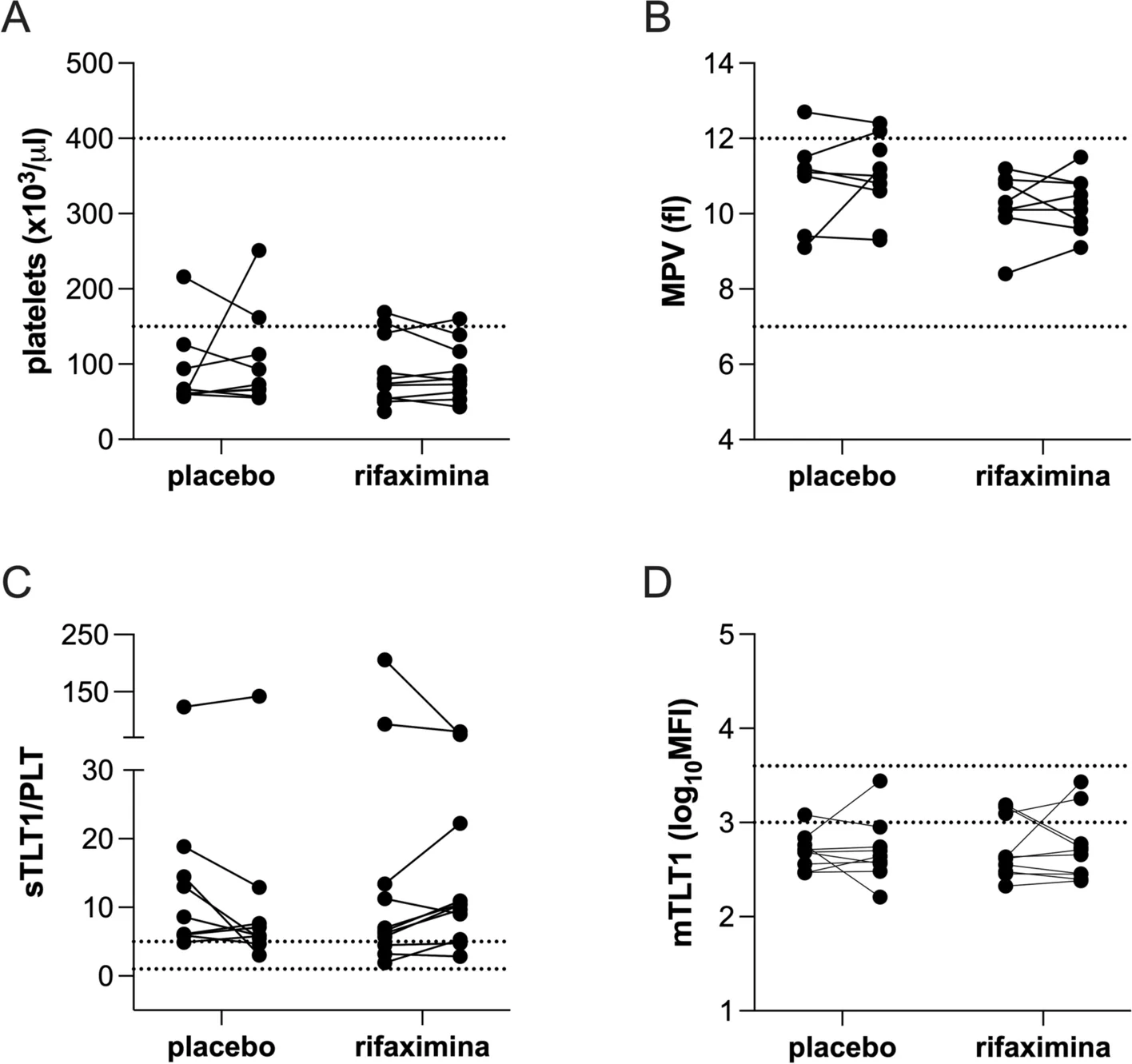
Platelet number, size, and activation before and after short-term therapy with rifaximin in decompensated cirrhosis patients. Before–after graph of **A** the platelet count, **B** the mean platelet volume (MPV), **C** the concentration soluble ectodomain of the TLT1 receptor, normalized to the platelet count (sTLT1/PLT), and **D** the log10 of the mean fluorescence intensity (log10MFI) of TLT1 exposed on the plasma membrane of platelets (mTLT1) in decompensated patients with cirrhosis randomized to receive either placebo (*n* = 9) or 550 mg of rifaximin (*n* = 12) for 14 days. Statistical significance was determined with a Multiple Wilcoxon test and no comparison was significantly different

Consistently, we did not detect any change in the platelet parameters in the entire cohort compared to the baseline values (Fig. 4). The platelet number did not change, and we did not observe any significant change in the platelet volume, in the surface levels of TLT-1 or other receptors (Supplementary Fig. 2) and on the plasma levels of soluble TLT-1.

## Discussion

The main findings of this study are that 1) patients with decompensated cirrhosis display markers of gut dysbiosis, chronic inflammation, NETosis, and platelet pre-activation in the systemic circulation; 2) the distinctive feature of circulating platelets in decompensated cirrhosis is the shedding of the platelet–megakaryocyte receptor TLT-1; and 3) short-term therapy with a non-absorbable antibiotic is not sufficient to reduce the inflammatory and prothrombotic state of these patients.

The hemostatic balance across the spectrum of chronic liver disease has been investigated extensively to understand the mechanisms behind the coexistence of bleeding and thrombosis at the end-stage of liver diseases [19]. Previous work demonstrated an association between markers of systemic inflammation such as IL-6 and the neutrophil–lymphocyte ratio and subsequent development of portal vein thrombosis (PVT) [20]. Neutrophils, the first responders to injury and infections, can be highly thrombogenic [21], as they release neutrophil extracellular traps (NETs), web-like structures of chromatin and granular proteins with procoagulant and platelet-activating effects. Previous studies have implicated neutrophils in the development of chronic liver disease [22], and NETs markers have been found to be higher in cirrhosis patients with PVT compared to patients without PVT and to correlate with markers of coagulation [23].

When it comes to the involvement of primary hemostasis, studies on platelet function in patients with liver cirrhosis have provided conflicting results [9, 24–31], possibly because many relied on ex vivo functional tests which can be affected by the low platelet count and by the doses of the exogenous agonists used [9]. To overcome the pitfalls and drawbacks of prior works, we here exploit flow cytometry that can investigate multiple parameters of platelet function and phenotype in a very low volume of blood and independently of the platelet count [15]. To ensure future reproducibility of the data and minimize artifacts, we did not add any exogenous agonist and performed the sample labeling and fixation within 30 min from blood withdrawal. We measured the surface levels of 13 platelet receptors, including adhesion receptors that mediate homotypic (e.g., PEAR1, CD41) or heterotypic (e.g., TLT1, CD40L, P-selectin) interactions and signaling receptors that induce either inhibitory (CD31) or stimulatory (e.g., GPVI, TLR4, CLEC2, CD32) pathways. Some of the detected receptors (TLT1, CD40L, and P-selectin) are stored in the alpha-granules of quiescent platelets, become exposed to the surface after activation and then are cleaved after ligand engagement. Our data showed that surface TLT-1 was significantly reduced on the platelet plasma membrane, while plasma levels of the soluble ectodomain of TLT1 were increased compared to age-matched controls. These data, together with the elevated PDW (i.e., a measure of platelet size heterogeneity), suggest that circulating platelets of decompensated patients with cirrhosis are preactivated in vivo as they have already mobilized their granules. Thus, our data are in line with previous studies that measured lower levels of intracellular ATP and hypothesized that platelets of patients with cirrhosis are less responsive ex vivo due to pre-activation and exhaustion of the intracellular stores [32]. However, our data are an important confirmation because intracellular ATP is measured ex vivo and can be affected by sample manipulation. Although previous studies detected high levels of the soluble ectodomain of CD40L and P-selectin, we did not detect lower levels of these receptors on the surface of platelets from our cohort. The reason for this inconsistency may be that CD40L and P-selectin are also expressed and cleaved from the surface of other cell types, while TLT-1 is expressed solely in the platelet–megakaryocyte lineage and is considered a more sensitive marker of platelet-specific activation than P-selectin [33]. Elevated levels of soluble TLT-1 are an indicator of poor prognosis in patients with acute lung injury and acute respiratory distress syndrome [34]. Thus, future studies could investigate the potential of using sTLT1 as an easy-to-measure biomarker to improve diagnosis and prognosis of liver disease patients. Furthermore, our data also identify the lower expression of PEAR1 as a distinct feature of circulating platelets in decompensated cirrhosis. GWAS studies identified PEAR1 as one of the top genes associated with platelet responsiveness and aggregability [35]. Thus, it would be interesting to investigate the correlation between PEAR1 surface expression with cardiovascular risk in liver disease patients.

Beyond defining a more detailed phenotype of platelet in cirrhosis, we also investigated whether platelet function would have responded to an intervention targeting the bacterial translocation and the increase of LPS, typical feature of cirrhosis, more so of decompensated stages, and recognized as main driver of platelet hyperactivation [9]. The first RCT investigating the role of rifaximin in decreasing LPS was conducted by Bajaj Js et al. in 2013, which showed that a dose of rifaximin 550 mg BID for 8 weeks was able to reduce endotoxin levels compared with baseline [36]. Twenty patients with decompensated cirrhosis were analyzed after 4 weeks of treatment with rifaximin 400 mg TID. Again, the use of rifaximin was associated with a reduction in whole blood endotoxin activity compared with baseline [37]. Several studies reported similar results with different doses or durations of therapy [38–40]. More recently, the largest RCT conducted by Patel V.C et al. administered rifaximin 550 mg BID for 30 and 90 days and showed a reduction in intestinal and systemic inflammation [41], leading the authors to conclude that rifaximin may improve bacterial translocation by promoting homeostasis in the intestinal barrier and microbiota.

While antibiotics consistently reduce the level of LPS in patients with advanced liver disease, few data were available in terms of modulation of the hemostatic balance in relation to its use. Previous work has shown how the use of non-absorbable antibiotics added to standard therapy for 7 days reduces levels of endotoxemia and markers of procoagulant status (i.e., prothrombin fragment F1 + 2 and D-dimer) compared with standard therapy alone in patients with cirrhosis [13].

In the present study, we conducted a pilot RCT in which a short-term treatment with rifaximin was insufficient to achieve the goal of reducing bacterial translocation and improving platelet function compared with placebo. We instead observed an improvement in bacterial translocation due to the reduction in LPS, not only in the rifaximin treated group but also in the placebo group. The platelet function remains unchanged, as do levels of inflammatory cytokines, while NETs were lower after treatment in both groups. These variations, together with the reduction in LPS levels, were likely associated in both groups to the best clinical practice adopted in the management of decompensated cirrhosis during the RCT, particularly in the treatment of ascites decompensation. Furthermore, as compared with prior works, the neutral effect of rifaximin was probably due to the shorter duration of treatment. Indeed, in the study by Violi et al. [13], an even shorter time was effective in modulating the hemostatic balance; however, the intervention was based on different non-absorbable antibiotics.

The strength of this study is that by exploiting flow cytometry, we quantify multiple parameters of the phenotype of circulating platelets in a minimal volume of blood and therefore identify markers of platelet pre-activation that are independent of the platelet count in patient with cirrhosis that have thrombocytopenia as major feature of the clinical syndrome. Moreover, the pilot study was conducted with randomization and double-blind design.

Nevertheless, our findings should be cautiously interpreted in light of several limitations. Firstly, the small size of the study population limited the analysis of possible confounding factors and the role of etiology in modulating platelet activity, as well as the discrimination of other determinants of liver disease in the results. Secondly, the small number of patients limited the ability to assess the impact of best clinical practice on the results compared to an active drug. However, the number of patients analyzed is compatible with the design of pilot studies. Furthermore, the short treatment duration can partially explain the null effect of rifaximin. It cannot be excluded that a longer treatment of at least four weeks with rifaximin would have revealed an effect on platelet phenotype. Finally, circulating LPS levels are also influenced by other factors, such as intestinal barrier resilience, immune clearance, and the presence of covert infections that were not systematically investigated in this study. Nevertheless, they are useful and easy-to-measure markers in the context of a pilot study.

In conclusion, the low-inflammatory state, NETosis and platelet pre-activation are key features of decompensated cirrhosis. Short-term therapy with rifaximin was ineffective in improving these abnormalities in addition to those resulting from the application of best clinical practice. Further analyses with a longer treatment period are needed to explore in depth the effect of non-absorbable antibiotics on the modulation of bacterial translocation, systemic inflammation, and platelet function in relation to clinical outcomes in patients with decompensated cirrhosis.

## Supplementary Information

Below is the link to the electronic supplementary material.

Supplementary file1 (TIFF 300 KB)

Supplementary file2 (TIFF 296 KB)

Supplementary file3 (PDF 390 KB)

## Data Availability

The data that support the findings of this pilot randomized controlled trial are not publicly available due to privacy and ethical restrictions, but are available from the corresponding author on reasonable request and with institutional review board approval.

## Abbreviations

CP: Child–Pugh
GPVI: Glycoprotein VI
HCC: Hepatocellular carcinoma
HE: Hepatic encephalopathy
LPS: Lipopolysaccharide
PEAR1: Platelet endothelial aggregation receptor 1
RCT: Randomized controlled trial
SAE: Serious adverse event
sTLT-1: Soluble TREM-like transcript-1
TLR4: Toll-like receptor 4
TLT-1: TREM-like transcript-1
