# Supplementary material for: Platelet activation and short-term rifaximin therapy in decompensated cirrhosis: a pilot randomized controlled study
Source: Intern Emerg Med. 2026 May 29;21(6):2141–52. doi: 10.1007/s11739-026-04399-7 (PMC13582090; doi:10.1007/s11739-026-04399-7)
Supplement: Supplementary file 1 [file 11739_2026_4399_MOESM3_ESM.pdf]

Platelet Activation and Short-term Rifaximin Therapy in Decompensated Cirrhosis: a Pilot Randomized Controlled Study

Authors

Simone Di Cola[1]§, Francesca Maiorca[1]§, Lucia Stefanini[1], Annamaria Sabetta[1], Ludovica Lombardi[1], Tania D'Amico[1], Lucia Lapenna[1], Valentina Castellani[1-2], Giulio Francesco Romiti[3], Roberto Cangemi[1], Oliviero Riggio[1], Valeria Raparelli[1]*, Manuela Merli[1]#, Stefania Basili[1]#

§*Co-first authorship*

#*Co-last senior authorship*

**Corresponding author*

Affiliations

[1] Department of Translational and Precision Medicine, Sapienza University of Rome, Rome, Italy

[2] Department of Medical and Surgical Sciences and Biotechnologies, Sapienza University of Rome, Latina, Italy

[3] Department of Wellbeing, Health and Environmental Sustainability, Sapienza University of Rome, Rieti, Italy

Address for Correspondence

*Valeria Raparelli, MD, PhD

Sapienza University of Rome

Department of Translational and Precision Medicine,

Viale dell'Università 37

00185 Rome, Italy

Email: valeria.raparelli@uniroma1.it

Supplementary Methods

Platelet phenotypic and functional analysis

For the platelet phenotypical characterization, citrated whole blood diluted 1:10 with Tyrode's buffer, containing mM (137 NaCl, 0.34 Na_2HPO_4, 2.9 KCl, 12 $NaHCO_3$, 10 N-2-hydroxyethylpiperazine-N'-2-ethanesulfonic acid, 5 D-glucose, 0.35% bovine serum albumin) pH 7.3 was incubated with saturating concentrations of α-CD41, α-CD62P, α-GPVI, α-CD42b, α-CD40L, α-TLT-1, α-CLEC2, α-CD36, α-PEAR1, α-CD32, α-TLR4, α-CD44 or α-CD31 (BD Bioscience), fixed with 1% formaldehyde, acquired on a BD Accuri C6 Plus, and analyzed with the FlowJo LLC software. The expression of each receptor was quantified based on the median fluorescence intensity among CD41+events.

Soluble TLT1 quantification

Plasma levels of the soluble ectodomain of the receptor TREML1/TLT1 (sTLT-1) were measured using a commercial immunoassay (Finetest). Detection was performed reading optical density at 450 nm by Multiskan SkyHigh Microplate Reader (Thermo Scientific). Plasma sTLT1 levels (pg/mL) were normalized to the platelet count to take into account the number of platelets from which sTLT1 was released.

Characterization of systemic inflammation

Serum cytokine concentrations were assessed using the LEGENDplex™ bead-based immunoassay (Inflammatory cytokines panel I; BioLegend) following manufacturer's specifications. Briefly, serum was diluted 1:2 in the assay buffer and incubated with fluorescence encoded beads and cytokine-specific antibodies, which allow simultaneous quantification of 13 human inflammatory cytokines and chemokines, including interleukin (IL)-1β, interferon (IFN)-α2, IFN-γ, tumor necrosis factor (TNF)-α, IL-6, IL-8, IL-10, IL-12p70, IL-17A, IL-18, IL-23, IL-33, and monocyte chemoattractant protein (MCP)-1. Six thousand events in the FSC/SSC gate that identified the beads were acquired with a BD Accuri C6 Plus and the data were analyzed with the LEGENDplex™ Data Analysis Software Suite (Version 2024-06-15. © 2019 Qognit). The concentration of each cytokine expressed in picogram/milliliter (pg/mL) was calculated based on the fluorescence intensity of each bead by sigmoidal interpolation of a standard curve.

Quantification of serum markers of neutrophil extracellular traps

Myeloperoxidase-DNA complexes were used as surrogate markers of neutrophil extracellular traps (NETs) and detected by capture ELISA, based on Kessenbrock et al[19]. Briefly, 5 μg/mL of anti--MPO antibody (Biorad), was used to coat a 96--well microtiter plate overnight at 4°C. After blocking with 1% BSA, sera was added at a 1:3 dilution to each well and was incubated for 1 hour at room temperature. After washing, peroxidase--labelled anti--DNA monoclonal antibody (component No. 2 of the Cell death detection ELISA kit, Roche) was added for 2 hours at room temperature and detected by adding the peroxidase substrate according to the manufacturer's instructions.

Quantification of serum LPS

LPS serum levels were measured with a commercial enzyme-linked immunosorbent assay kit (Cusabio). An antibody specific for LPS was precoated onto a microplate, and 100μl of standards or samples were incubated for 2 hours at room temperature. After incubation, samples were read at 450 nm using Multiskan SkyHigh Microplate Reader (Thermo Scientific). Values were expressed as pg/mL; intra-assay and interassay coefficients of variation were 8% and 10%, respectively.

Supplementary Figure 1

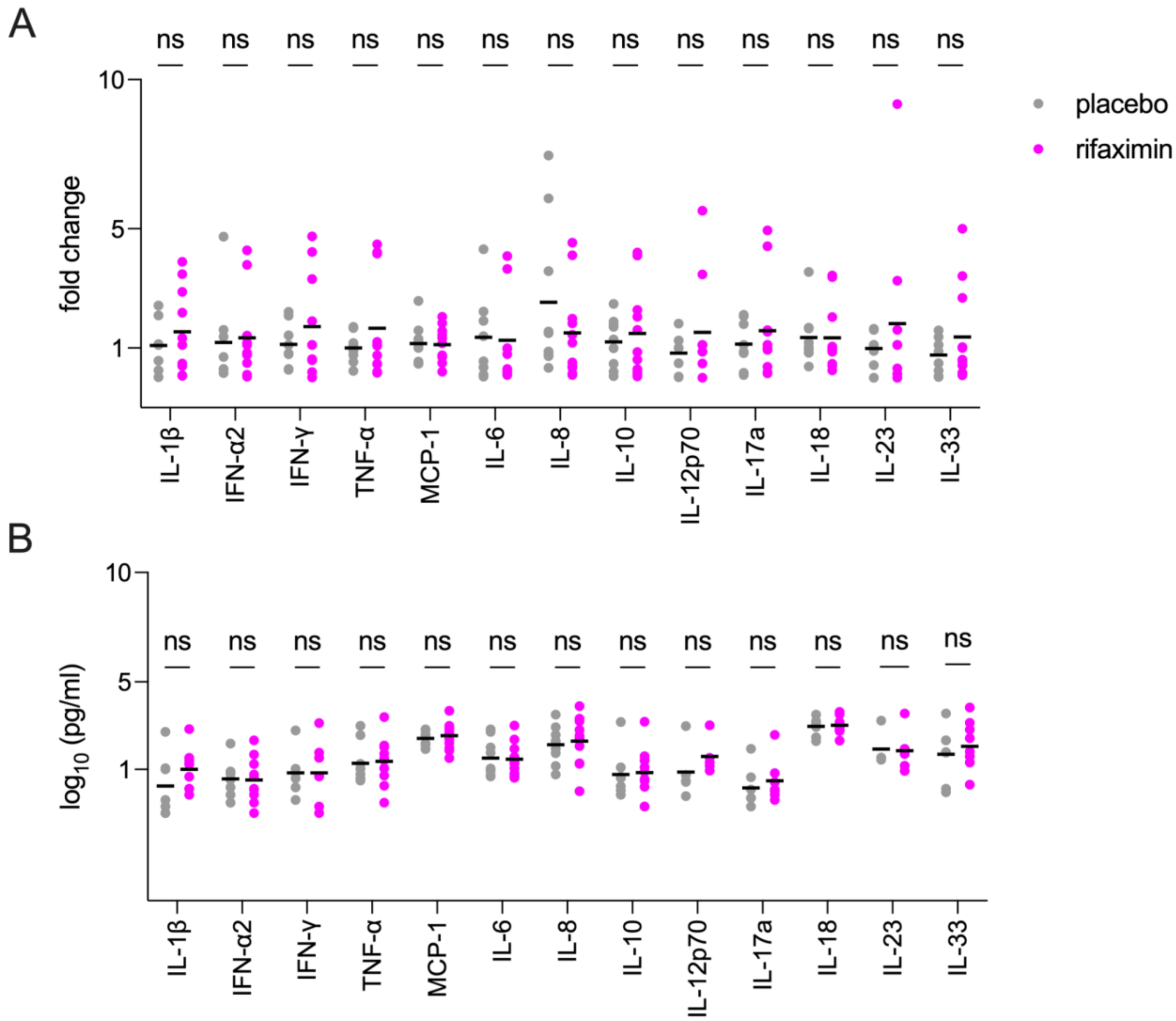

Multiple Mann Whitney t test of the fold change (**A**) and $\log_{10}$ (pg/ml) (**B**) of the concentration of inflammatory cytokines in decompensated cirrhotic patients randomized to receive either placebo (n=9, grey) or 550 mg of rifaximin (n=12, pink) for 14 days. The graph shows the fold change between the median fluorescence intensity (MFI) after and before treatment of varius interleukins (IL), Tumor Necrosis Factor-alpha (TNF-α) and Interferon alpha-2 (IFN-α2). The line indicates the median value. The Holm-Šídák method was used to adjust for multiple comparisons; * adjusted $p<0.05$.

Supplementary Figure 2

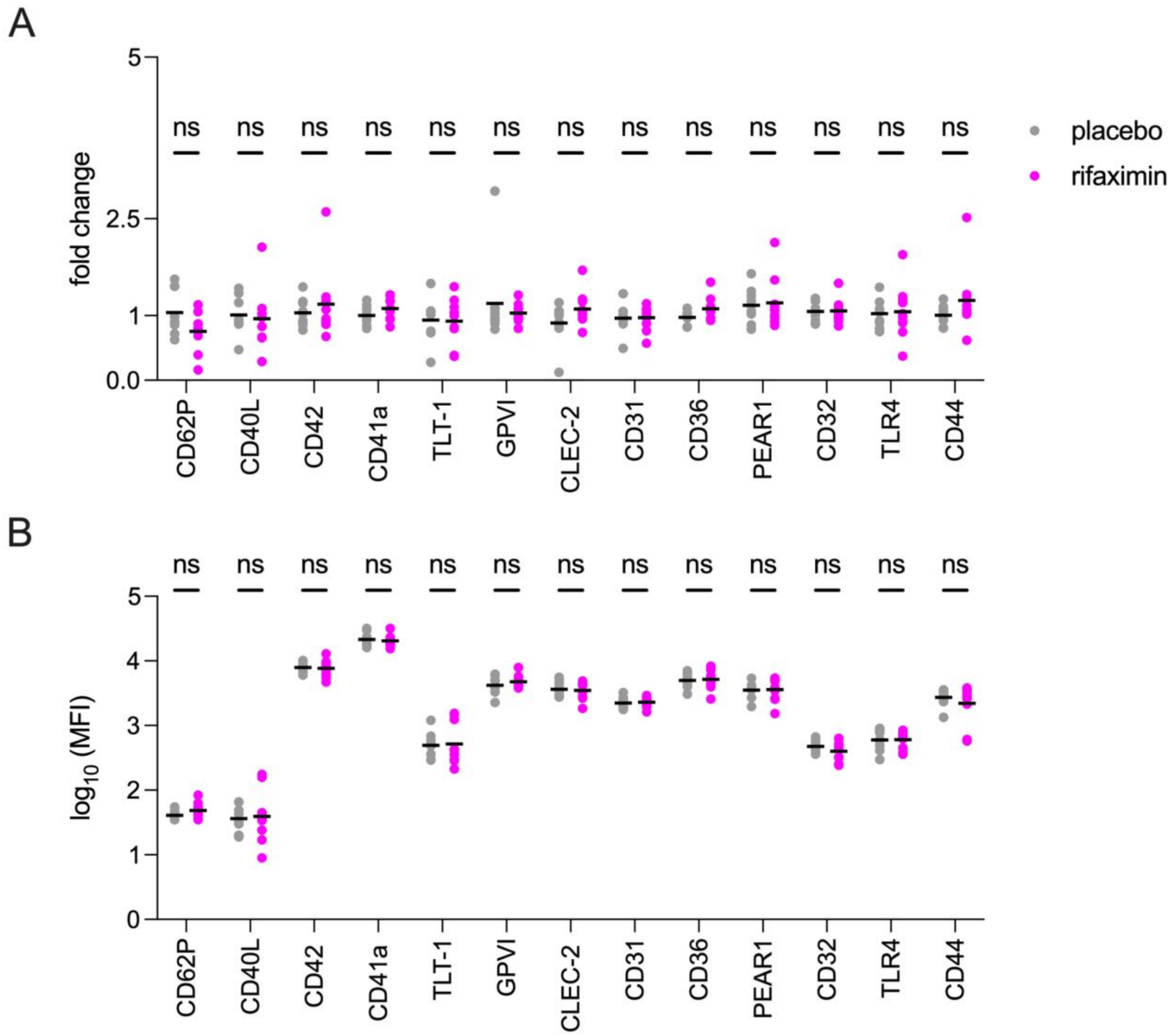

Multiple Mann Whitney t test of the fold change (**A**) and log_{10} (MFI) (**B**) of 13 platelet surface receptor expression levels (detected by flow cytometry) in decompensated cirrhotic patients randomized to receive either placebo (n=9, grey) or 550 mg of rifaximin (n=12, pink) for 14 days. The graph shows the fold change between the median fluorescence intensity (MFI) after and before treatment of P-selectin (CD62P), CD40 ligand (CD40L), glycoprotein Iba (CD42b), integrin aIIb (CD41a), TREM-like 1 (TLT-1), glycoprotein VI (GPVI), C-Type Lectin Domain Family 1 Member B (CLEC2), PECAM-1 (CD31), CD36, Platelet Endothelial Aggregation Receptor 1 (PEAR1), FcγRIIa (CD32), Toll-like receptor 4 (TLR4), CD44. The line indicates the median value. The Holm-Šídák method was used to adjust for multiple comparisons; * adjusted p<0.05.
